# Recipients with *In Utero* Induction of Tolerance Upregulated MHC Class I in the Engrafted Donor Skin

**DOI:** 10.1155/2014/531092

**Published:** 2014-07-20

**Authors:** Jeng-Chang Chen, Liang-Shiou Ou, Hsiu-Yueh Yu, Ming-Ling Kuo, Pei-Yeh Chang, Hsueh-Ling Chang

**Affiliations:** ^1^Department of Surgery, Chang Gung Children's Hospital, College of Medicine, Chang Gung University, 5 Fu-Shin Street, Kweishan, Taoyuan 333, Taiwan; ^2^Department of Allergy, Asthma and Rheumatology, Chang Gung Children's Hospital, College of Medicine, Chang Gung University, 5 Fu-Shin Street, Kweishan, Taoyuan 333, Taiwan; ^3^Pediatric Research Center, Chang Gung Children's Hospital, 5 Fu-Shin Street, Kweishan, Taoyuan 333, Taiwan; ^4^Department of Microbiology and Immunology, Graduate Institute of Biomedical Sciences, College of Medicine, Chang Gung University, 259 Wen-Hwa 1st Road, Kweishan, Taoyuan 333, Taiwan

## Abstract

The alterations in MHC class I expression play a crucial step in immune evasion of cancer or virus-infected cells. This study aimed to examine whether tolerized grafts modified MHC class I expression. FVB/N mice were rendered tolerant of C57BL/6 alloantigens by *in utero* transplantation of C57BL/6 marrows. Postnatally, engrafted donor skins and leukocytes were examined for their MHC expression by quantitative real-time PCR and flow cytometry. Engrafted donor skins upregulated their MHC class I related gene transcripts after short-term (1~2 weeks) or long-term (>1 month) engraftment. This biological phenomenon was simultaneously associated with upregulation of TAP1 gene transcripts, suggesting an important role of TAP1 in the regulation of MHC class I pathway. The surface MHC class I molecules of H-2K^b^ in engrafted donor leukocytes consistently showed overexpression. Conclusively, the induction of allograft tolerance involved biological modifications of donor transplants. The overexpression of MHC class I within engrafted transplants of tolerant mice might be used as the tolerance biomarkers for identifying a state of graft tolerance.

## 1. Introduction

Organ transplantation has been the standard practice for failed or failing organs. However, long-term graft survivals rely upon immunosuppressive therapies that lessen or prevent rejection episodes at the cost of direct organ toxicity, susceptibility to opportunistic infections, malignancy, and accelerated cardiovascular diseases [1, 2]. Despite a barely imagined concept in the past, achieving tolerance to transplanted organs is the best way to solve the problems imposed by immunosuppressive therapies. Thus, modern transplantation medicine is moving away from the exploration of novel immunosuppressive agents toward the induction of allograft tolerance in organ recipients. It causes a pressing need to search for unique tolerance biomarkers as surrogate endpoints of immunosuppressive strategies, or measures for predicting successful tolerance induction [2, 3]. Recipients with graft tolerance retain well-functioning grafts after cessation of immunosuppressive regimens, referred to as operational tolerance [4]. They are the best proof of concept for immunosuppressive-free graft survivals and the potential candidates for the quest of tolerance signatures [1, 5–7]. However, this unique paradigm of human tolerance develops exclusively under the aegis of antecedent immunosuppressive therapies. The effects of immunosuppressive cessation on these recipients remain a matter of concern to the interpretation of tolerance biomarkers generated [1, 8]. Several lines of evidence have revealed a critical role for MHC class I (MHC-I) molecules in immune evasion of virus-infected or cancer cells [9, 10], suggesting that tolerized grafts might have to undergo similar biological alterations. In this tolerance model without the employment of immunosuppression, engrafted donor skins upregulated MHC-I associated gene transcripts and engrafted donor cells also overexpressed surface MHC-I molecules. Thus, graft tolerization facilitated MHC-I expression within donor grafts.

## 2. Materials and Methods

### 2.1. Mice

FVB/N (H-2^q^) mice were used as recipients, C57BL/6 (H-2^b^) as donors, and C3H (H-2^k^) as the source of third-party alloantigens. They were purchased at their age of 6–8 weeks and bred for this study with the approval of the Committee on Animal Research at Chang Gung Memorial Hospital. Recipient females were caged with males in the afternoon and checked for vaginal plugs the following morning. The day when the plug was observed was designated as day 0 of the pregnancy.

### 2.2. Preparation of Donor Cells and* In Utero* Transplantation

C57BL/6 bone marrow cells (BMCs) were harvested by flushing the tibias and femurs with phosphate buffer saline (PBS) using a 26-gauge needle, layered over NycoPrep 1.077A (Nycomed, Pharma AS, Oslo, Norway), and centrifuged at 600 g for 25 minutes. Light-density BMCs were then depleted of T-cells by anti-CD3*ε* FITC (BioLegend, San Diego, CA) and anti-FITC microbeads (Miltenyi Biotec, Auburn, CA). T-cell-depleted BMCs contained CD3^+^ cells of <0.5% by flow cytometry.

Donor BMCs were freshly injected within 3 hours after preparation. Briefly, the uteri of gestational day 14 pregnant FVB/N mice were exposed through a vertical laparotomy. A 60 *μ*m glass micropipette with beveled tip was used to inject 5–10 × 10^6^ T-cell-depleted BMCs in 5–10 *μ*L of PBS into the peritoneal cavities of all fetuses at a litter via transuterine approach. Then, the abdomen of the pregnant mice was closed in two layers with 5–0 silk. After operation, all mice were housed in an undisturbed room without bedding changes until the pups were 1 week old. Pups were weaned at 3 weeks of age.

### 2.3. Analyses of Chimerism and MHC-I Expression of Donor Leukocytes

Chimerism levels were examined at the age of 1 month old. Peripheral blood was sampled via tail veins and depleted of red cells using ACK lysing buffer. Cells were first incubated with anti-mouse Fc*γ*II/Fc*γ*III antibody (BioLegend) and then stained with anti-H-2K^q^ FITC (BioLegend) and anti-H-2K^b^ PE (BioLegend). Chimerism levels were determined by flow cytometry after gating out dead cells by high propidium iodide staining. A negative control consisted of anti-H-2K^q^ FITC and mouse IgG2a PE (BioLegend) to define background staining. Recipients with first month peripheral chimerism of >3% were collected to further validate tolerance by skin transplantation because they could be consistently rendered tolerant to donor skin [11]. As for the quantification of donor leukocyte's MHC-I expression, engrafted donor leukocytes were gated, and the fluorescence intensities (FIs, including mean, geographic mean, and median) of anti-H-2K^b^ PE were measured. Wild-type C57BL/6 mice were used as controls.

### 2.4. Skin Transplantation

Under anesthesia, the recipient's back was shaved by clippers and disinfected by beta-iodine solution. Graft beds with intact panniculus carnosus were created by the scissors [12]. Skin transplants from tails were placed on the graft bed, fixed by stitches, covered by Vaseline gauze, and dressed by Band-Aid. After the removal of dressings on day 7, grafts were monitored daily. Skin tolerance was defined by donor skin engraftment with good hair growth for at least 4 months. Engrafted donor skin was subjected to histological examinations after hematoxylin-eosin staining in order to exclude the possibility of microscopic rejection.

### 2.5. mRNA Extraction from Skin Grafts of Tolerant Mice

The engrafted skin grafts were taken down from tolerant mice at indicated time points. Then, their cell constituents were homogenized by physical treatment using a tissue tearor in Trizol Reagent (Invitrogen, USA). mRNAs were extracted with RNeasy Mini Kit (Qiagen K.K., Tokyo, Japan) according to the instruction manual. The purity of RNA samples was evaluated using absorption of light at 260 and 280 nm (A260/280) by a ND-2000 spectrophotometer (Nanodrop Technology, USA). High-quality mRNA showed the A260/280 ratio of ≥1.97.

### 2.6. Quantitative Real-Time PCR for MHC Expression of Skin Grafts

First-strand cDNA were reversely transcribed from mRNA templates of each skin sample in equal amount, using MMLV High Performance Reverse Transcriptase Kit with an oligo(dT) primer (EPICENTRE Biotechnologies, Madison, WI). Then, MHC-I related target gene expression was quantified by Bio-RAD iQ5 real-time PCR detection system, using Maxima SYBR Green/ROX qPCR Master Mix (2X) (Fermentas, Thermo Scientific). The specificity of real-time PCR reaction was further confirmed by melting curve analysis. Normalized values for target mRNA expression in each sample were calculated as the relative quantity of target gene divided by the relative quantity of glyceraldehyde-3-phosphate dehydrogenase (GAPDH). All the gene primers were designed by online primer design tool of Primer 3 as shown in Table 1.

### 2.7. Statistical Analyses

The equality of means was examined by Student's *t*-test between two independent or paired-sample groups. Differences were regarded as significant in case of *P* < 0.05.

## 3. Results

### 3.1. Screening of MHC Gene Expression in Engrafted Donor Skins

**Figure 1:**
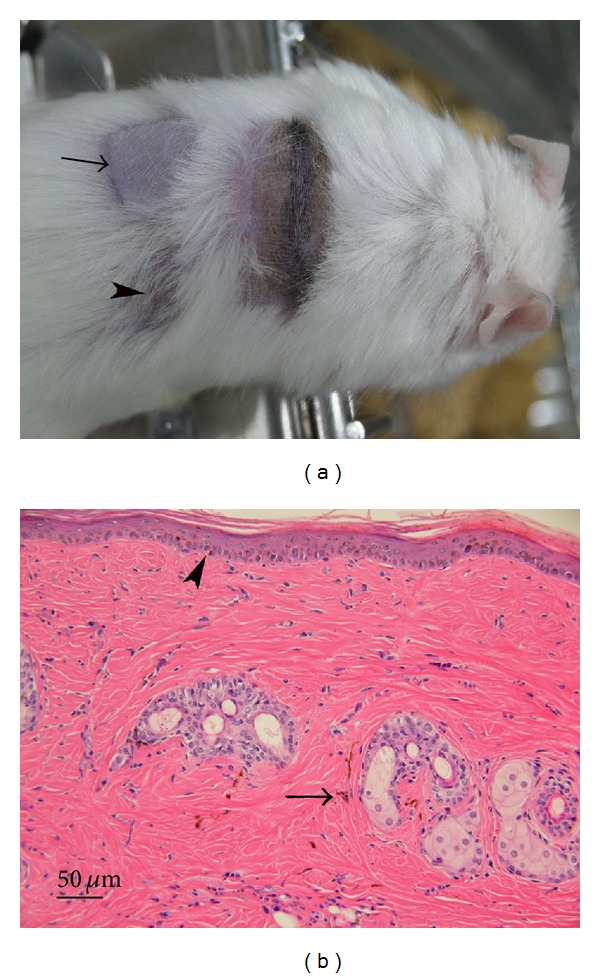
Donor-specific skin tolerance. Mixed chimerism was established in FVB/N mice through* in utero* transplantation of C57BL/6 marrows. A representative FVB/N mixed chimera was rendered tolerant to C57BL/6 donor skin (a). The donor-specific (black hair) and syngeneic (arrow) skins had been accepted for more than 4 months after their placement. The rejection of third-party C3H skin caused an area of scarring (arrowhead). Hematoxylin-eosin staining did not reveal inflammatory cell infiltration in the engrafted C57BL/6 donor skin (b), which contained melanin pigments within keratinocytes of basal epidermis (arrowhead) and in the dermis (arrow).

**Figure 2:**
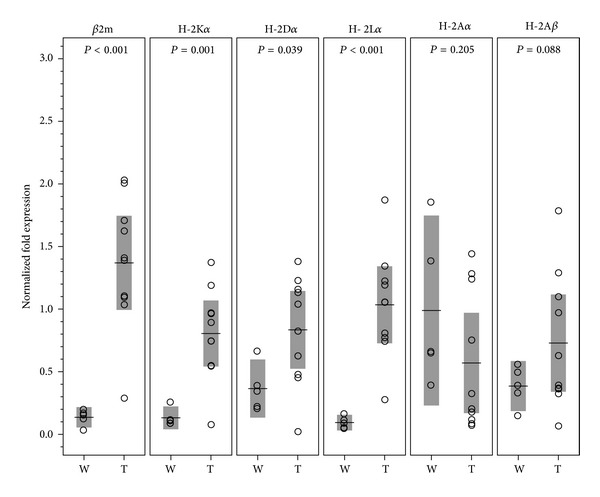
The expression of MHC related genes within engrafted C57BL/6 donor skins. MHC-I genes including *β*2m, H-2K*α*, H-2D*α*, and H-2L*α* were upregulated in engrafted donor skins (T) as compared with wild-type donor skins (W). However, the expressions of MHC class II (MHC-II) H-2A*α* and H-2A*β* in engrafted and wild-type C57BL/6 skins were comparable. MHC-II H-2E is nonfunctional in C57BL/6 mice and not tested. The means and their 95% confidence intervals are shown as horizontal lines and shade boxes, respectively.

We collected 13 mixed chimeras with first month donor cell (C57BL/6) levels of 6.05~28.18%. All were readily rendered tolerant to donor skin for at least 4 months (Figure 1). Engrafted donor skins had no histological evidence of inflammatory cell infiltration (Figure 1). Following mRNA extraction, 10 samples showed high-quality mRNA with A260/280 of ≥1.97. The remaining 3 samples were disqualified due to a lower A260/280 ratio of 1.84~1.92. Extracted mRNA from 5 wild-type C57BL/6 skins had the A260/280 ratio of ≥1.97. Quantitative real-time PCR was then performed following reverse transcription from mRNA of each sample in equal amount. The mRNA expression of *β*2m, H-2K*α*, H-2D*α*, H-2L*α*, H-2A*α*, and H-2A*β* genes was determined by normalized fold expression relative to the mRNA quantity of GAPDH internal control gene. This survey for MHC gene expression revealed that engrafted donor skins significantly upregulated *β*2m, H-2K*α*, H-2D*α*, and H-2L*α* as opposed to wild-type controls (Figure 2).

### 3.2. Gene Expression of Accessory Proteins in MHC-I Processing Pathway

**Figure 3:**
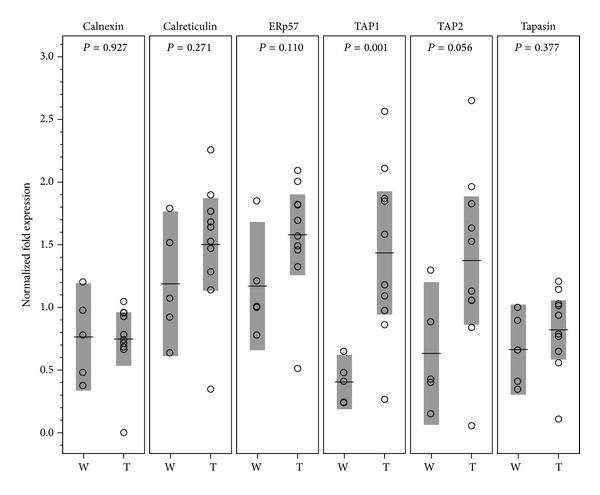
The mRNA expressions of accessory proteins in MHC-I pathway. As compared with wild-type donor skins (W), engrafted donor skins (T) had significant upregulation of TAP1 (*P* = 0.001), but only borderline upregulation of TAP2 (*P* = 0.056). The means and their 95% confidence intervals are shown as horizontal lines and shade boxes, respectively.

We further examined the expression of mRNA that translates accessory proteins responsible for assembly and transport of MHC-I molecules. These chaperone-like proteins in MHC-I processing pathway include the transporter associated with antigen processing (TAP1 & TAP2), tapasin, calnexin, calreticulin, and ERp57. Engrafted donor skins showed significant upregulation of TAP1, but only marginal upregulation of TAP2 (Figure 3).

### 3.3. MHC-I and TAP Gene Expression in Donor Skins before and after Transplantation

**Figure 4:**
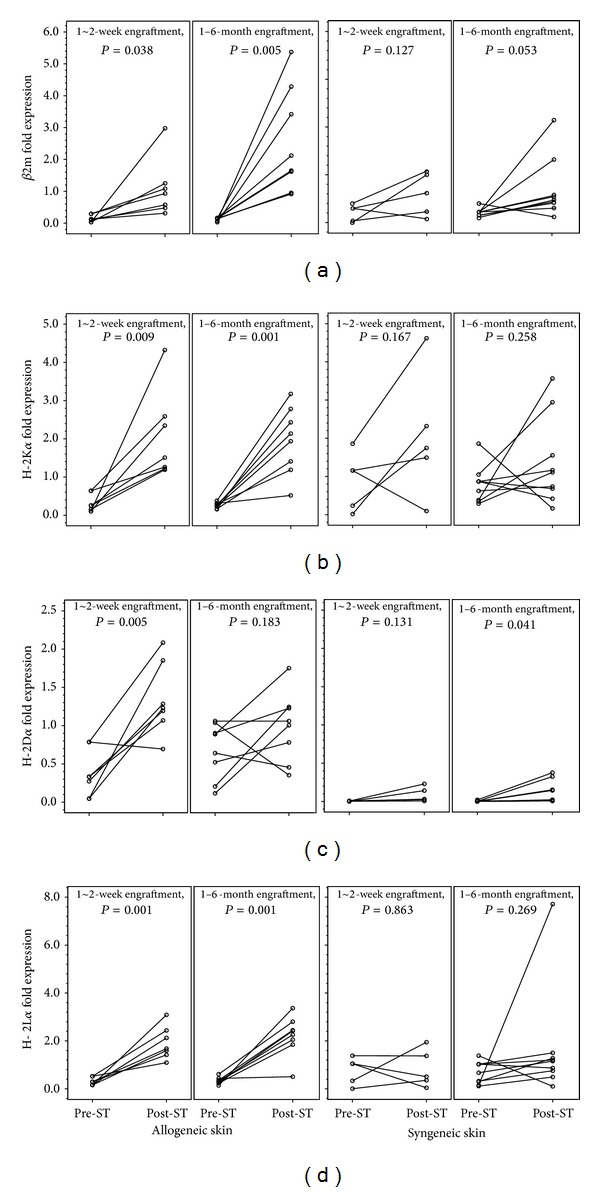
MHC-I upregulation in the process of donor skin tolerization. MHC-I related gene expression of donor skins was examined before (Pre-ST) and after (Post-ST) skin transplantation. The upregulation of *β*2m (a), H-2K*α* (b), H-2D*α* (c), and H-2L*α* (d) in donor skins showed up at the beginning of engraftment (1~2 weeks, *n* = 7) in tolerant mice and remained so within 1~6 months (*n* = 8) after skin transplantation except for H-2D*α*. Syngeneic donor skins did not exhibit the similar upregulation of MHC-I related genes after short-term (1~2 weeks, *n* = 5) and long-term (1~6 months, *n* = 9) engraftment.

**Figure 5:**
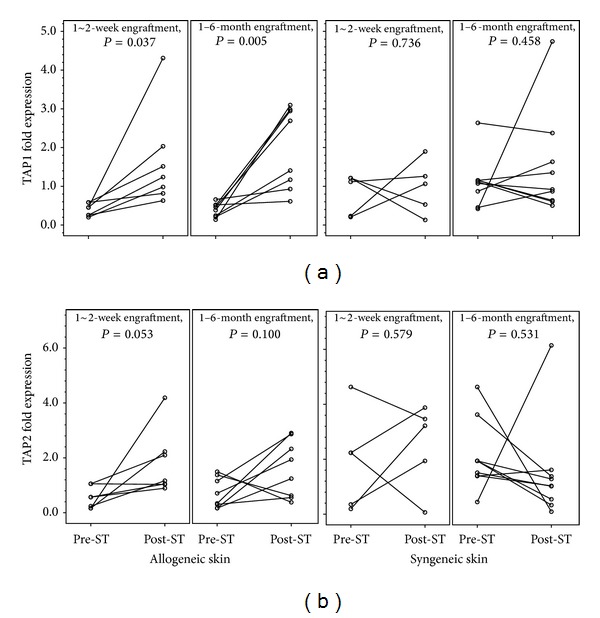
TAP1 and TAP2 expression in the process of donor skin tolerization. The engraftment of donor skins in tolerant recipients, either short-term (*n* = 7) or long-term (*n* = 8), enhanced the expression of TAP1 (a) rather than TAP2 (b) within donor skins. Neither TAP1 nor TAP2 was consistently upregulated within syngeneic skins after short-term (*n* = 5) and long-term (*n* = 9) engraftment.

For further validating the upregulation of MHC-I related gene transcripts within tolerized grafts, we performed the matched-pairs studies for MHC-I (*β*2m, H-2K*α*, H-2D*α*, and H-2L*α*) and TAP1&2 gene expressions of skin grafts before and after their transplantation. The mice with skin tolerance were used as recipients for secondary skin transplantation after removal of prior engrafted skins. Both allogeneic (C57BL/6) and syngeneic (FVB/N) tail skins were first collected before their placement on tolerant mice. Following skin transplantation, the engrafted skin graft from the same donor tail was harvested at indicated time points (1~2 weeks and >1 month). Following mRNA extraction (A260/280 ratio ≥1.97) and reverse transcription, the expressions of target genes were measured in relation to those of an endogenous GAPDH reference gene by quantitative real-time PCR. Paired-sample *t*-test was used to compute the differences between values of the two normalized expression variables for each skin graft from the same donor before and after skin transplantation (pre-ST and post-ST). The expressions of *β*2m, H-2K*α*, H-2L*α*, and TAP1 were significantly increased in donor skins after short-term (1~2 weeks) and long-term (>1 month) engraftment. However, H-2D*α* was only significantly upregulated in short-term engrafted donor skin (Figures 4 and 5). Syngeneic skin grafts did not exhibit significant upregulation of MHC-I related genes after engraftment.

### 3.4. Surface MHC-I Expression of Donor Leukocytes in Tolerant Mixed Chimeras

**Figure 6:**
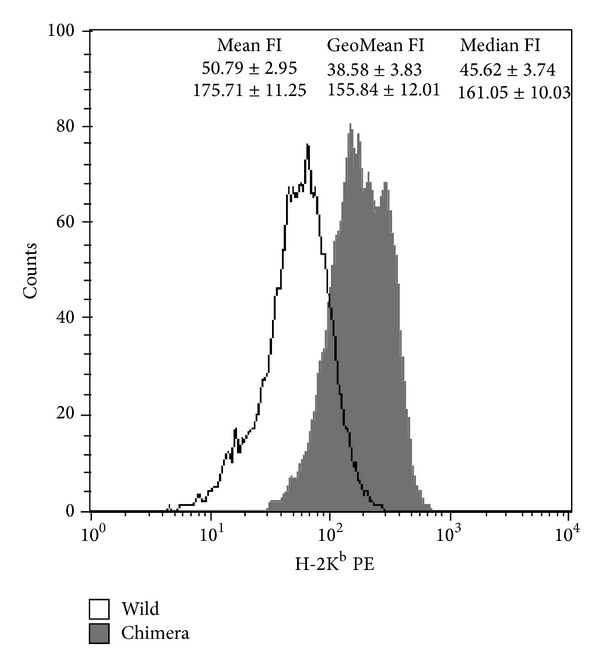
Surface H-2K^b^ FIs of engrafted donor leukocytes in 6-month-old mixed chimeras with skin tolerance. This representative histogram showed surface H-2K^b^ PE FIs of C57BL/6 donor leukocytes from a mixed chimera and an age-matched wild-type C57BL/6 control. Chimeras (*n* = 5) had higher mean, geographic mean, and median H-2K^b^ FIs than wild-type C57BL/6 controls (*n* = 5). Data were expressed as mean ± standard error of mean. *P* < 0.001 for mean, geographic mean (GeoMean), and median FIs.

Surface MHC-I protein expression on engrafted donor leukocytes was quantified by flow cytometry. Five tolerant mixed chimeras with donor cell levels of 4.28~35.12% at their age of 6 months were collected for the evaluation of H-2K^b^ mean, geographic mean, and median FIs. In tolerant mice, engrafted donor leukocytes had an about 3~4-fold increment of FIs as opposed to wild-type leukocytes (Figure 6).

## 4. Discussion

The first challenge that researchers face on initiating tolerance biomarker studies lies in the recruitment of subjects with operational tolerance [2, 13]. Human recipients can be sometimes rendered tolerant to transplanted kidneys [5, 8] or livers [6]. In renal recipients, either gradual or abrupt withdrawal of immunosuppressive regimens may trigger episodes of acute rejection. This inevitably causes certain degree of irreversible renal parenchymal damage to jeopardize the function and longevity of renal allografts [1]. Although long-term sequelae from liver graft rejection may be minimal or even absent due to strong liver regenerative capacity, it is viable only when liver rejection episodes can be detected and treated early after immunosuppressive minimization or discontinuation [1]. Thus, it is not advisable to intentionally taper or discontinue immunosuppressive regimens in recipients for scrutinizing an existing state of operational tolerance.

Clinically, operational tolerance is usually a serendipitous clinical event, occurring in 10–20% of renal [14] and liver [15] recipients. This small fraction of recipients were usually recognized under a special situation wherein they did well after immunosuppressive discontinuation as a result of their medical noncompliance, or clinical necessity such as life-threatening infection, malignancy, or unacceptable drug toxicity [1]. With the advent of high throughput biotechnology [16], these fortuitous recipients had been collected to profile their gene or proteome expression patterns [17]. The profiling of gene or proteome expressions requires appropriate controls for smoothing comparison so that researchers can identify specific variations attributed to a state or biological process of operational tolerance. However, operationally tolerant recipients were highly selected cases that had been universally subjected to long-term immunosuppressive therapies with diverse and complicated clinical courses. This made it difficult or even impossible to find appropriate control groups. In their simplest form, researchers may find the biomarkers that suffice to discriminate tolerance from nontolerance recipients. However, it is hard to ensure that the biomarkers are not merely a test to identify tolerance patients who are not taking immunosuppressive regimens [8]. Thus, it is important to enroll healthy cases given immunosuppressive regimens with and without subsequent immunosuppressive discontinuation so that we can analyze the contribution of immunosuppressive drugs or transplants to the profiles of assay results. However, it is merely a scientifically but not ethically sound approach. Despite this insurmountable obstacle in human subject studies for tolerance biomarkers, several studies had related operational tolerance to the alterations of natural killer (NK), regulatory (Treg), B- or *γ* *δ* T-cell associated gene expressions [4, 8, 18].

The discovery of trivial donor leukocytes in the tissues or blood of long-surviving organ recipients had once led to the notion that graft tolerance was linked by a common dependence on the presence of donor cell chimerism [19, 20]. It raised the expectations for using donor cell chimerism as an ideal tolerance biomarker. However, subsequent animal [21, 22] and human [23] studies showed that graft tolerance could persist in the absence of donor cell chimerism. In addition, graft rejection even occurred in a state of donor cell chimerism [24, 25]. This conflict might be ascribed to the emerging consensus that hematopoietic chimerism was relevant to the induction rather than the maintenance phase of graft tolerance [11, 26]. Despite the unreliability of donor cell chimerism as a tolerance biomarker [27, 28], the creation of mixed chimerism remains the most popular strategy to facilitate allograft tolerance in various animal models for the investigation of immune tolerance [29, 30]. Surprisingly, there are few such animal studies seeking to identify tolerance biomarkers [1]. It is likely due to the notion that the results obtained in animal studies may not always apply to humans. However, animal studies remain the important touchstones of many biological phenomena in humans. The establishment of mixed chimerism to facilitate graft tolerance through conventional marrow transplantation necessitates myeloablative or immunosuppressive therapies to modify the host immunity [20, 31]. Under the circumstances, tolerance biomarker studies still have to confront the interference of immunosuppressive therapies. In this regard,* in utero* marrow transplantation that requires no myeloablation and immunosuppression has unsurpassed advantage over conventional approach for tolerance-related studies. Thus, upregulation of MHC-I within engrafted transplants represented a biological phenomenon that was independent of immunosuppressive effects on the recipients.

In the pursuit of tolerance biomarkers, researchers mostly focused on the recipients in preference to the donor grafts for examining various biological activities or products that sufficed to signify a tolerant state. However, local regulatory mechanisms might contribute to graft tolerance due to Treg activity specifically within tolerated grafts [32, 33]. Evidence also showed that Treg-associated gene expressions in recipients destined for graft tolerance and rejection primarily differed within the transplanted grafts themselves rather than systemically or in spleens and draining lymph nodes of recipients [18]. As a result, graft tolerance might not necessarily elicit a unique biomarker circulating systemically but rather be an immunological event occurring locally within the target transplants. Thus, it makes sense to search for tolerance biomarkers in tolerated grafts or cells despite the fact that biomarkers are preferably measurable in readily accessible sources such as hosts' blood. Allogeneic skins engrafted either short-term or long-term exhibited upregulation of MHC-I associated transcripts as opposed to syngeneic skin grafts. Thus, MHC-I upregulation did not result from the procedure of skin transplantation itself, but rather the biological modifications of donor allografts in the process of graft tolerance. A simultaneous upregulation of TAP1 gene transcripts suggested a kinetically critical role of TAP1 in the regulation of MHC-I pathway. MHC-I protein expression was quantified on engrafted donor leukocytes by flow cytometry. Engrafted donor cells consistently overexpressed surface H-2K^b^ (MHC-I).

Discovering a biomarker for transplantation tolerance is not necessarily equivalent to uncovering their causal relationship. However, our approach to the secondary donor skin acceptance in tolerant recipients suggested that MHC-I overexpression was the recipients' tolerizing effects on donor grafts. Within the donor grafts, inflammatory responses might upregulate their MHC-I and related genes. Graft inflammation usually resulted from alloimmune reactions. However, the absence of mononuclear cell infiltration reflected the lack of any ongoing inflammatory process within the engrafted donor skins. Thus, MHC-I upregulation should be specific to tolerance rather than the results of alloimmunity. We have known that the alterations in MHC-I expression play a crucial step in immune evasion of cancer or virus-infected cells through the regulation of adaptive T-cell cytotoxicity [34] and innate NK cell function [35]. Most cancer [9] or virus-infected cells [10] downregulate MHC-I to escape adaptive T-cell cytolysis. In contrast, multidrug-resistant human cancer cell lines and flaviviruses such as hepatitis C virus may upregulate MHC-I in parallel with TAP1 to evade innate NK cell attacks for the establishment of multidrug resistance [36] and chronic hepatitis C infection [37]. Given the critical role of NK cells in the defense against virus-infected, neoplastic, and allogeneic cells [38], the inhibition of NK cell cytotoxicity from the interaction of MHC-I ligands and NK inhibitory receptors [39, 40] may be related to the establishment of graft tolerance and awaits further experimental elucidation in the future.

## 5. Conclusions

Immune tolerance to donor transplants involved biological modifications of MHC-I upregulation within engrafted donor grafts. This biological phenomenon has great potential to be a tolerance biomarker in the settings of transplantation. More importantly, it may pave the way to further mechanism studies for transplantation tolerance in the future.

## Figures and Tables

**Table 1:** Primers used for quantitative real-time PCR.

Gene	Forward primer	Reverse primer
GAPDH	TCACCACCATGGAGAAGGC	GCTAAGCAGTTGGTGGTGCA
*β*2m	TGGTGCTTGTCTCACTGACC	CCGTTCTTCAGCATTTGGAT
H-2K*α*	ACATGGAGCTTGTGGAGACC	TGTTGGAGACAGTGGATGGA
H-2D*α*	GCGGAGAATCCGAGATATGA	AGCCAGACATCTGCTGGAGT
H-2L*α*	GGAAAAGGAGGGGACTATGC	CAAGCTCACAGGGAACATCA
H-2A*α*	GAGTCACACCCTGGAAAGGA	ACAGCCTCAGGGTCAAGAGA
H-2A*β*	GGGTCTCATCCACACAGCTT	ACATTTTGCTCCAGGCAGAC
Calnexin	GGCTAGACGACGAACCTGAG	AGGCTTCCATTTGCCCTTAT
Calreticulin	GACTTTCTGCCACCCAAGAA	TCCCACTCTCCATCCATCTC
ERp57	TATGATGGGCCTAGGACTGC	TGCTGGCTGCTTTTAGGAAT
TAP1	CATCACATCTCGGGTGACTG	TGCACTTTTCCCAGCTTCTT
TAP2	GCTCCCTTTCAATGCCAATA	CACTGCATCCTGGATCTCCT
Tapasin	ACCTGGCTACGGTACACCTG	TCTGAGCTCCCACTTGACCT
